# Intracellular Staphylococcus aureus Modulates Host Central Carbon Metabolism To Activate Autophagy

**DOI:** 10.1128/mSphere.00374-18

**Published:** 2018-08-08

**Authors:** Natalia Bravo-Santano, James K. Ellis, Luis M. Mateos, Yolanda Calle, Hector C. Keun, Volker Behrends, Michal Letek

**Affiliations:** a Health Sciences Research Centre, University of Roehampton, London, United Kingdom; b Division of Cancer, Department of Surgery and Cancer, Faculty of Medicine, Imperial College London, London, United Kingdom; c Department of Molecular Biology, Area of Microbiology, University of León, León, Spain; University of Nebraska Medical Center

**Keywords:** Staphylococcus aureus, autophagy, host cell, intracellular pathogen, metabolism

## Abstract

Staphylococcus aureus is a facultative intracellular pathogen that invades and replicates within many types of phagocytic and nonphagocytic cells. During intracellular infection, S. aureus is capable of subverting xenophagy and escaping to the cytosol of the host cell. Furthermore, drug-induced autophagy facilitates the intracellular replication of S. aureus, but the reasons behind this are unclear. Here, we have studied the host central carbon metabolism during S. aureus intracellular infection. We found extensive metabolic rerouting and detected several distinct metabolic changes that suggested starvation-induced autophagic flux in infected cells. These changes included increased uptake but lower intracellular levels of glucose and low abundance of several essential amino acids, as well as markedly upregulated glutaminolysis. Furthermore, we show that AMP-activated protein kinase (AMPK) and extracellular signal-regulated kinase (ERK) phosphorylation levels are significantly increased in infected cells. Interestingly, while autophagy was activated in response to S. aureus invasion, most of the autophagosomes detected in infected cells did not contain bacteria, suggesting that S. aureus induces the autophagic flux during cell invasion for energy generation and nutrient scavenging. Accordingly, AMPK inhibition halted S. aureus intracellular proliferation.

**IMPORTANCE** Staphylococcus aureus escapes from immune recognition by invading a wide range of human cells. Once the pathogen becomes intracellular, the most important last resort antibiotics are not effective. Therefore, novel anti-infective therapies against intracellular S. aureus are urgently needed. Here, we have studied the physiological changes induced in the host cells by S. aureus during its intracellular proliferation. This is important, because the pathogen exploits the host cell’s metabolism for its own proliferation. We find that S. aureus severely depletes glucose and amino acid pools, which leads to increased breakdown of glutamine by the host cell in an attempt to meet its own metabolic needs. All of these metabolic changes activate autophagy in the host cell for nutrient scavenging and energy generation. The metabolic activation of autophagy could be used by the pathogen to sustain its own intracellular survival, making it an attractive target for novel anti-infectives.

## Abstract (precis)

Staphylococcus aureus escapes from immune recognition by invading a wide range of human cells. Once the pathogen becomes intracellular, the most important last resort antibiotics are not effective. Therefore, novel anti-infective therapies against intracellular S. aureus are urgently needed. Here, we have studied the physiological changes induced in the host cells by S. aureus during its intracellular proliferation. This is important, because the pathogen exploits the host cell’s metabolism for its own proliferation. We find that S. aureus severely depletes glucose and amino acid pools, which leads to increased breakdown of glutamine by the host cell in an attempt to meet its own metabolic needs. All of these metabolic changes activate autophagy in the host cell for nutrient scavenging and energy generation. The metabolic activation of autophagy could be used by the pathogen to sustain its own intracellular survival, making it an attractive target for novel anti-infectives.

## INTRODUCTION

The Gram-positive bacterium Staphylococcus aureus is a well-known opportunistic pathogen, thought to be carried by about one-third of the global human population on the skin and/or in the nasal passages (1, 2), which act as a reservoir for infections of the lower respiratory tract (3). S. aureus is considered one of the leading causes of hospital-acquired infections, although the number of community-associated S. aureus infections has also increased in recent decades (4).

While S. aureus was originally considered an extracellular pathogen (5), it has since been shown to be able to invade both phagocytic and nonphagocytic mammalian cells (6–9). Mechanistically, invasion of non-professional phagocytes by S. aureus is achieved via a zipper-type mechanism, involving fibronectin-binding proteins A and B (FnBPA and FnBPB) (10–12). Several bacterial factors such as wall teichoic acids (WTAs), protein A, and clumping factor B (ClfB) have also been shown to be important for host cell invasion (9). Once S. aureus is internalized, it is able to persist and replicate within phagosomes and, eventually, escape to the cytosol (9, 13), leading to the activation of host cell death mechanisms such as apoptosis (14, 15).

Effective invasion and proliferation of intracellular pathogens are directly connected to the metabolism of the host cell since the intracellular compartment in which the pathogen resides becomes the space from which it imports nutrients in order to survive and replicate (16, 17). Therefore, once bacteria have been internalized, both bacteria and the host cell share—and compete for—the same nutrients (18, 19). Intracellular pathogens have developed different mechanisms to acquire nutrients from the host (18), by either altering host metabolic pathways (20), raising nutrient import (21), or exploiting/subverting host mechanisms to degrade macromolecules such as autophagy (22).

Autophagy is a catabolic mechanism that involves the formation of double-membrane vesicles—autophagosomes—and subsequent lysosomal fusion to degrade damaged or undesirable cytosolic material (23, 24). It is a well-conserved pathway in eukaryotic cells and plays important physiological roles in response to nutrient starvation, physiological stress, and recycling of organelles (24–26). Despite their names, (auto)phagosomes are also involved in a common host response against intracellular bacteria called xenophagy (27). It is known that a number of intracellular pathogens, including S. aureus, can subvert xenophagy to persist within the autophagosomes before escaping to the cytosol (28). However, it is not yet fully understood how S. aureus achieves this and which host pathways and/or metabolites it uses to enhance its intracellular survival and/or replication.

Treatment of S. aureus infections is significantly complicated by the ability of the pathogen to establish intracellular infection (29) and thus evade large parts of the host’s immune response, particularly if the emergence of multidrug-resistant strains, such as methicillin-resistant S. aureus (MRSA), is taken into account (30, 31). MRSA is resistant to many of the first-line antibiotics traditionally used to treat Gram-positive bacteria, and the three “last resort” antibiotics routinely employed to treat MRSA infection (vancomycin, daptomycin, and linezolid) are unable to enter the cell in sufficient quantities to achieve intracellular killing (32). Therefore, there is an urgent need to find novel therapies against this versatile pathogen. One strategy is to identify and target host pathways essential for pathogen survival and proliferation (33, 34).

Our aim for this study was to understand the host cell metabolic changes induced by MRSA infection with a view to identifying novel anti-infective strategies against MRSA infections. We show that MRSA infection leads to changes in the metabolic fluxes of the host cell. These changes lead to a starvation-like state in the host cell and the subsequent activation of autophagy.

## RESULTS

### The extracellular metabolome of MRSA-infected host cells.

**FIG 1:**
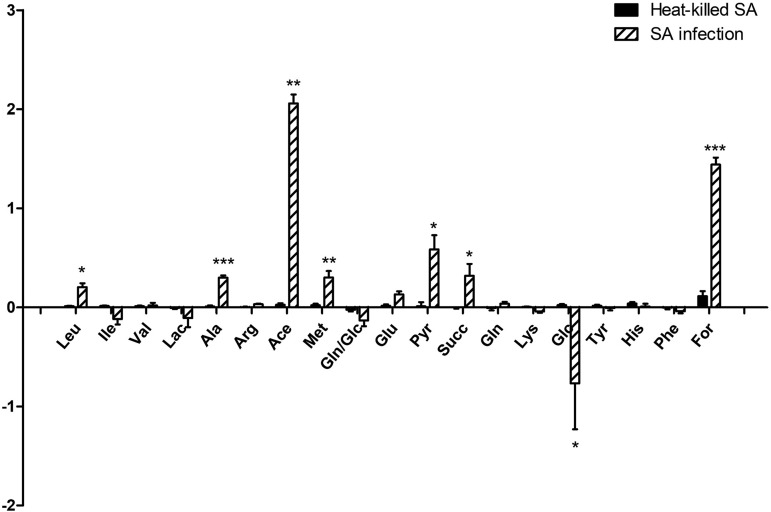
Comparison of metabolites identified in the media after MRSA invasion. HeLa cells were exposed to live and heat-killed USA300 cells (MOI of 100) for 6 h, and medium composition was analyzed by NMR spectroscopy. Results are shown as log_10_ fold change relative to the uninfected condition, and the figure shows means ± standard errors from two independent experiments performed in triplicate. Striped bars and black bars represent cells infected with live and heat-killed USA300 cells, respectively. Statistical differences were tested using Student’s *t* test against uninfected cells. *, *P* ≤ 0.05; **, *P* ≤ 0.01; ***, *P* ≤ 0.001.

We used nuclear magnetic resonance (NMR) spectroscopy to analyze the medium composition during gentamicin protection assays in HeLa cells exposed to both live and heat-killed S. aureus USA300 cells. Results showed an increased concentration of organic acids in the medium of infected cells with viable USA300 cells, such as formic, pyruvic, and succinic acids. In particular, the highest increase was detected for acetic acid, with an increase of more than 100-fold compared to uninfected cells (Fig. 1). Levels of some amino acids (leucine, alanine, and methionine) were also increased in the medium of infected cells (Fig. 1). In contrast, glucose levels were significantly reduced compared to those in the uninfected control (Fig. 1), suggesting more rapid glucose uptake. No significant changes were found in the medium of cells infected with heat-killed USA300, suggesting that the increased secretion of organic acids and the lower levels of glucose were resulting from metabolic changes in the host cell triggered by viable intracellular bacteria.

### Changes in the host cell metabolism in response to MRSA infection. (i) Changes in the central carbon metabolism after S. aureus USA300 infection.

**FIG 2:**
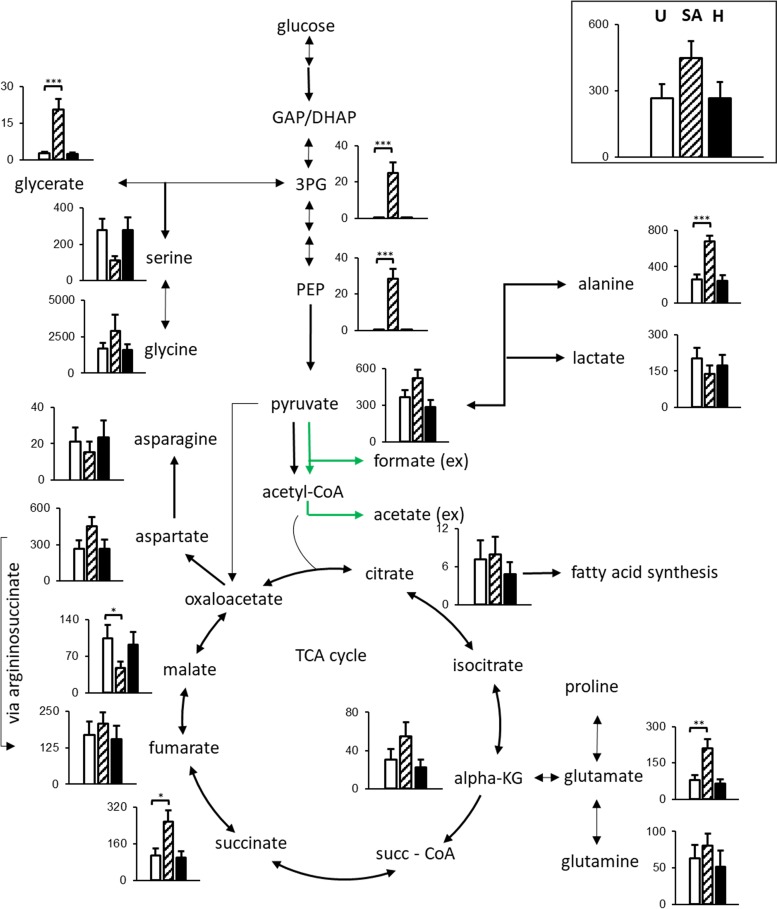
Differences in the pool sizes of metabolites of the central carbon metabolism after S. aureus USA300 infection in HeLa cells. HeLa cells were lysed after gentamicin protection assays with both live and heat-killed USA300 cells, and metabolites were detected by GC-MS. Graphs show absolute levels of each metabolite in uninfected cells (U [white bars]), cells infected with the USA300 strain (SA [striped bars]), and cells exposed to heat-killed USA300 (H [black bars]). The figure shows means ± standard errors from two independent experiments performed in triplicate. Statistical differences were tested using Student’s *t* test against uninfected cells. *, *P* ≤ 0.05; **, *P* ≤ 0.01; ***, *P* ≤ 0.001. The *y* axis scale units are arbitrary units (×1,000). Green arrows indicate metabolites of microbial origin.

**FIG 3:**
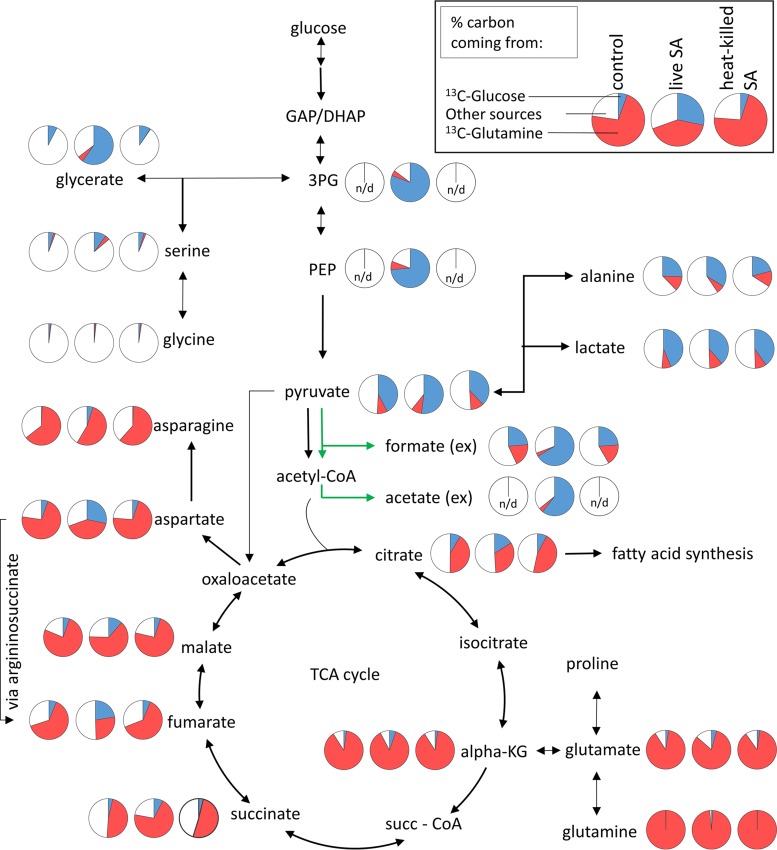
Differences in the labeling patterns of metabolites of the central carbon metabolism after MRSA infection. HeLa cells were infected with USA300 and exposed to heat-killed USA300 cells (MOI of 100) for 6 h. Metabolites and labeling distribution were obtained by GC-MS. The figure illustrates the labeling pattern of each metabolite in uninfected cells (left pie chart), cells infected with live USA300 cells (middle pie chart), and cells exposed to heat-killed USA300 cells (right pie chart). Pie charts depict the labeling distribution, where blue sections represent carbon coming from a glucose-labeled source, red sections represent carbon coming from a glutamine-labeled source, and white sections show carbon from other sources. Green arrows indicate metabolites of microbial origin.

The intracellular metabolome of MRSA-infected cells was analyzed by gas chromatography-mass spectrometry (GC-MS) (Fig. 2 and 3). We detected a major increase of several glycolytic intermediates during MRSA invasion. Levels of both 3-phosphoglycerate (3-PG) and phosphoenolpyruvate (PEP) were over 50 times higher in MRSA-infected cells compared to uninfected cells or cells exposed to heat-killed bacteria (Fig. 2). In addition, the glycerate pool size in the MRSA-infected cells significantly increased compared to that of controls (Fig. 2). The labeling distribution of glycerate (Fig. 3) suggests that it was formed from glycolytic 3-PG. In contrast, pyruvate levels only increased slightly in the MRSA-infected cells, whereas lactate levels remained mostly unchanged among the three conditions (Fig. 2).

Several tricarboxylic acid (TCA) cycle intermediates such as α-ketoglutarate, citrate, and succinate were present at higher levels in HeLa cells infected with USA300 (Fig. 2). Interestingly, this increase was not universal, as levels of fumarate remained unchanged among the three conditions and malate levels were reduced in cells infected with MRSA (Fig. 2). Further, stable isotope tracing suggested that most of the carbon of succinate is derived from glutamine; in contrast, the labeling patterns of fumarate and malate showed an enhancement of glucose-derived label, suggesting an increased importance of extramitochondrial pools of these metabolites. Interestingly, an increase in glucose-derived carbon can also be seen in aspartate (Fig. 3). For both fumarate and aspartate, this increase was due to a rise in the M+3 isotope (see Fig. S1 at https://figshare.com/s/e297ca2455dccb05307c).

### (ii) Amino acid metabolism.

We observed an activation of glutaminolysis in HeLa cells infected with USA300, with higher uptake of glutamine from the medium and unchanged intracellular levels. In contrast, glutamate and alanine levels were significantly higher in MRSA-infected cells than in uninfected cells or cells exposed to heat-killed MRSA (Fig. 2). In the case of alanine, there is a slight increase of glucose-derived label, indicating increased flux from pyruvate (Fig. 1 and 2).

**FIG 4:**
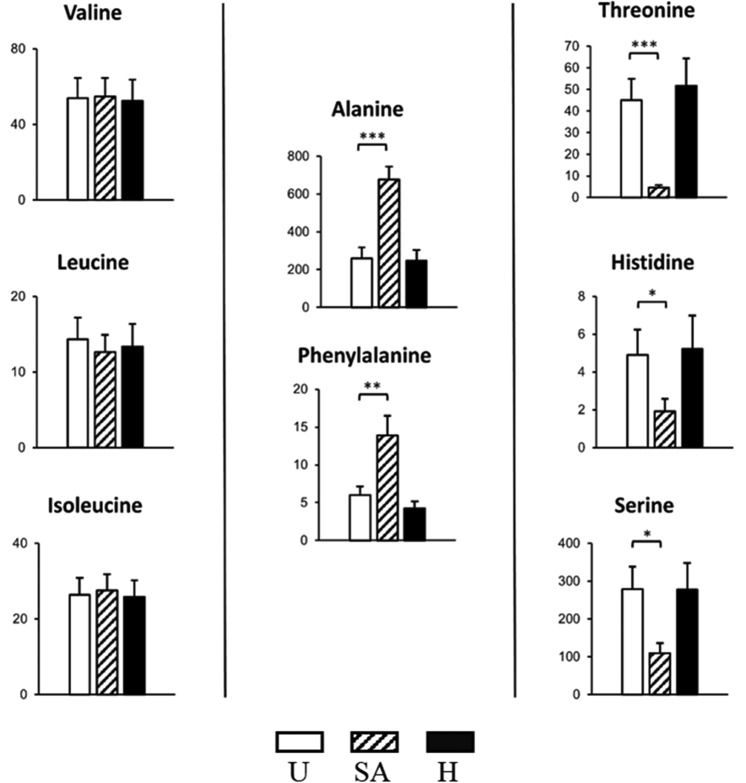
Changes in absolute levels of amino acids in HeLa cells after S. aureus USA300 infection. HeLa cells were infected with USA300 and exposed to heat-killed USA300 cells (MOI of 100) for 6 h. Levels of amino acids were detected and quantified by GC-MS. Graphs show absolute levels of each amino acid in uninfected cells (U [white bars]), cells infected with live USA300 cells (SA [striped bars]), and cells exposed to heat-killed S. aureus USA300 cells (H [black bars]). The figure shows means ± standard errors from two independent experiments performed in triplicate. Statistical differences were tested using Student’s *t* test against uninfected cells. *, *P* ≤ 0.05; **, *P* ≤ 0.01; ***, *P* ≤ 0.001. The *y* axis scale units are arbitrary units (×1,000).

Branched-chain amino acids are important for the virulence of S. aureus (35). However, we have observed no differences in the levels of valine, leucine, and isoleucine among the three experimental conditions (Fig. 4).

Importantly, the pool sizes of histidine, threonine, and serine (three essential amino acids for both S. aureus and HeLa cells) were significantly reduced after USA300 infection (Fig. 4). In contrast, phenylalanine levels of infected cells were also increased. While phenylalanine cannot be synthesized *de novo* by mammalian cells, MRSA is able to synthesize phenylalanine from phenylpyruvate and histidine via HisC (SAUSA300_0708), a histidinol-phosphate aminotransferase, potentially explaining both higher levels of phenylalanine and lower levels of histidine in MRSA-infected cells.

### (iii) Changes in the central carbon metabolism after vancomycin protection assays with S. aureus NCTC 13626.

The significance of certain host-pathogen interactions may vary depending on the cell line or bacterial strain tested (9). For example, the metabolism of cancer cell lines is conditioned by the Warburg effect (36); therefore, the metabolomics findings described in HeLa cells may not be applicable to primary cells. To address this, we studied the metabolism of HeLa cells and BALB/c mouse bone marrow macrophages when infected with MRSA strain NCTC 13626, a reference strain for the health care-associated MRSA sequence type 239 (HA-MRSA ST239) lineage (37, 38). NCTC 13626 is resistant to gentamicin; therefore, the infection assays were carried out in the presence of vancomycin. Interestingly, most of the changes induced in the metabolism of both types of host cells tested with NCTC 13626 were in concordance with the results observed in HeLa cells infected with USA300 (see Fig. S2 and S3 at https://figshare.com/s/e297ca2455dccb05307c). The levels of glucose were reduced in HeLa cells and bone marrow macrophages in response to NCTC 13626 infection (see Fig. S2 and S3), whereas glutamate levels were significantly increased, especially in bone marrow macrophages (Fig. S3), suggesting an activation of both the glycolysis and glutaminolysis pathways. The increased levels of glycolytic intermediates were found in HeLa cells irrespective of the infecting strain.

Similar to the USA300-infected cells, several metabolites from the TCA cycle such as citrate and α-ketoglutarate were significantly increased in NCTC 13626-infected cells compared to uninfected controls (see Fig. S2 and S3 at https://figshare.com/s/e297ca2455dccb05307c). The levels of succinate were also increased in HeLa cells infected with NCTC 13626 (Fig. S2), whereas succinate levels were stable in bone marrow macrophages (Fig. S3). In contrast with USA300 infection, levels both of fumarate and malate were higher in HeLa cells and bone marrow macrophages when infected with NCTC 13626, suggesting a strain-dependent metabolic change (see Fig. S2 and S3).

### Staphylococcal infection induces autophagy in HeLa cells.

A number of the metabolic changes we observed in this study have previously been linked to autophagy (i.e., an increase in glutaminolysis and low levels of glucose and essential amino acids may activate the autophagic flux in mammalian cells) (39). Further, autophagy/xenophagy is a common host response against intracellular infection, where the autophagosomes are employed to engulf intracellular bacteria for later degradation. However, different studies have proved that S. aureus is able to survive and replicate within these autophagosomes before escaping to the cytosol of the cell (9).

**FIG 5:**
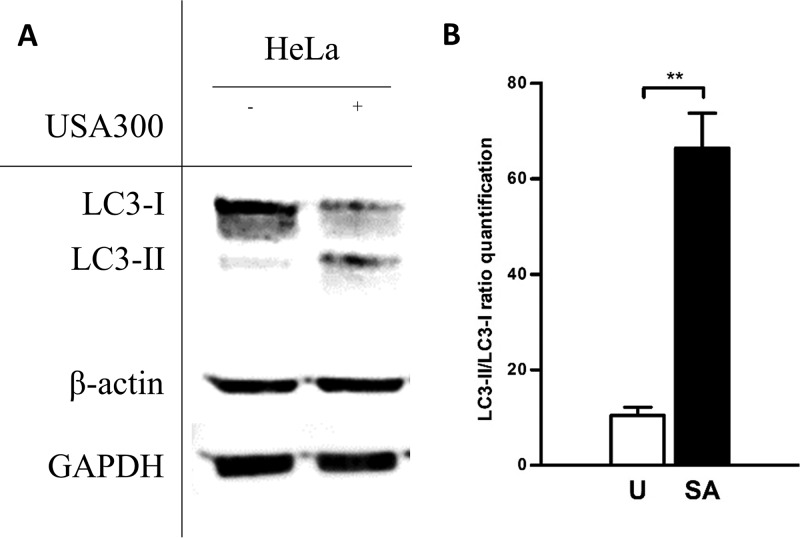
MRSA infection induces autophagy in HeLa cells. (A) HeLa cells were infected with the USA300 strain (MOI of 100) for 6 h, and protein lysates were analyzed by Western blotting against LC3. The figure shows that MRSA infection promotes autophagy by activating the conversion of LC3-I to LC3-II. Both actin and GAPDH antibodies were employed as loading controls. The figure is a representative example of three independent experiments. (B) Quantification of the conversion of LC3-I to LC3-II in uninfected and infected HeLa cells. Data are expressed as means ± standard errors from three different experiments, and *t* tests were performed to validate statistical significance across conditions. U (white bar), uninfected cells; SA (black bar), S. aureus-infected cells. **, *P* ≤ 0.01.

**FIG 6:**
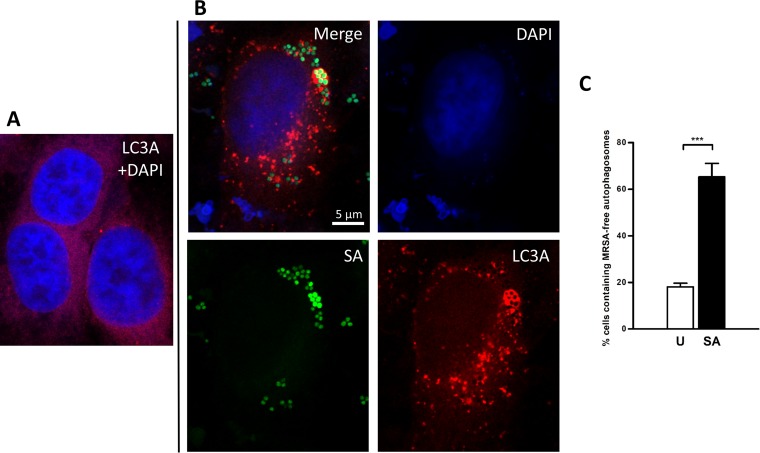
Colocalization assay of MRSA-GFP and mCherry-LC3A by confocal microscopy. (A) Uninfected HeLa cells expressing mCherry-LC3A. (B) HeLa cells expressing mCherry-LC3A infected with the USA300-GFP strain (MOI of 100 for 6 h); DAPI was employed for nucleus staining. The MRSA-free autophagosomes were detected around the whole cell. (C) Quantification of percentage of cells containing MRSA-free autophagosomes in uninfected (U [white bar]) and S. aureus-infected (SA [black bar]) HeLa cells. Data are expressed as means ± standard errors from three different experiments, and *t* tests were performed to validate statistical significance across conditions. ***, *P* ≤ 0.001.

We therefore investigated autophagy status in our infection model. The conversion of endogenous levels of LC3-I to LC3-II by Western blotting is a standard measure of autophagy activation (40). Accordingly, the levels of conversion of LC3-I to LC3-II are markedly increased in MRSA-infected HeLa cells compared to a noninfected control (Fig. 5). However, after careful analysis by confocal microscopy, we observed that cells with vacuoles staining positive for the autophagosomal marker mCherry-LC3 did not necessarily colocalize with bacteria (Fig. 6). Furthermore, 45% of intracellular bacteria were cytosolic at time point 6 h, which allows direct access to host metabolites present in the cytosol, such as glucose (see Fig. S4 at https://figshare.com/s/e297ca2455dccb05307c). As one of the main roles of autophagy is degradation of organelles for energy generation and nutrient scavenging (41), we have studied the phosphorylation state of AMP-activated protein kinase (AMPK) and extracellular signal-regulated kinase (ERK) during MRSA intracellular infection: AMPK and ERK are sensors of energy stress and amino acid deprivation in mammalian cells.

AMPK is a key regulator in cellular energy homeostasis in eukaryotes, and this pathway is activated when intracellular levels of ATP are low (42, 43). Furthermore, a drop of amino acids in eukaryotic cells has been linked to a stimulation of autophagy by activating the Ras/Raf1/ErkI/II pathway (44).

**FIG 7:**
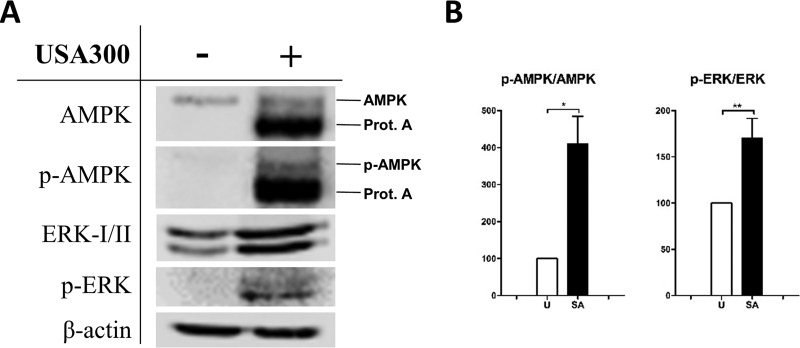
An activation of the p-AMPK and p-ERK2 pathway was detected in MRSA-infected cells. HeLa cells were infected with USA300 cells (MOI of 100) for 6 h. (A) Protein lysates were analyzed by Western blotting against AMPK, phosphorylated AMPK (p-AMPK), p44/42 MAPK (ErkI/II), and phospho-p42/44 AMPK (p-ERK). β-Actin antibody was employed as a loading control. (B) Quantification of the phospho-AMPK/AMPK and phospho-ErkI/ErkII ratios. Data were normalized to β-actin levels for each condition. Data are expressed as means ± standard errors from three different experiments, and *t* tests were performed to validate statistical significance across conditions. U (white bars), uninfected cells; SA (black bars), S. aureus-infected cells. *, *P* ≤ 0.05; **, *P* ≤ 0.01.

Therefore, we quantified the levels of AMPK and ErkI/II on MRSA-infected cells, and we found that both pathways were activated in response to bacterial infection in HeLa cells. Basal levels of ErkI/II and AMPK were increased in HeLa cells infected with MRSA; however, the levels of the phosphorylated versions of both proteins were even higher (Fig. 7A). Thus, ratios of phospho-AMPK/AMPK and phospho-ErkI/ErkII were, respectively, 4 and 1.7 times higher than those in the noninfected control (Fig. 7B).

**FIG 8:**
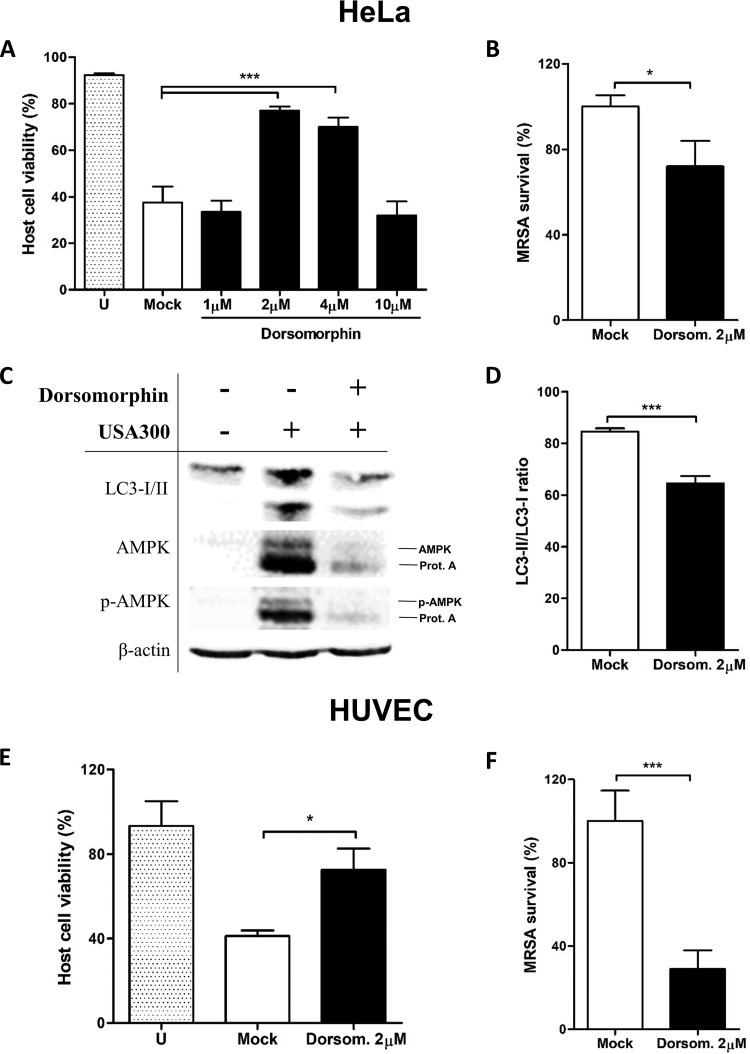
AMPK inhibition increases cell viability while reducing intracellular MRSA survival and autophagy levels. HeLa cells and HUVECs were infected with USA300 (MOI of 100) for 6 h in the presence or absence of dorsomorphin. (A and E) Host cell viability after USA300 infection was quantified by flow cytometry in HeLa cells and HUVECs, respectively. (B and F) intracellular MRSA survival was measured by CFU counting in agar plates in HeLa cells and HUVECs, respectively. (C) Protein lysates were analyzed by Western blotting against LC3I/II, AMPK, and phosphor-AMPK (p-AMPK). β-Actin antibody was employed as a loading control. (D) Quantification of the conversion of LC3-I to LC3-II. Data are expressed as means ± standard errors from three different experiments, and *t* tests were performed to validate statistical significance across conditions. U (stippled bars), uninfected. *, *P* ≤ 0.05; ***, *P* ≤ 0.001.

To further validate these results, we tested the effect of dorsomorphin—an AMPK inhibitor (45)—on MRSA infection in human umbilical vein endothelial cells (HUVECs) and HeLa cells. We first confirmed that dorsomorphin has no direct effect on USA300 growth (see Fig. S5 at https://figshare.com/s/e297ca2455dccb05307c). We then tested the effect of dorsomorphin on the cell viability of USA300-infected cells. High concentrations of dorsomorphin did not restore host cellular viability during infection, indicating that this drug can be cytotoxic at high doses to mammalian cells (Fig. 8A). However, in the presence of 2 µM dorsomorphin, host cell viability was increased in comparison to that of a nontreated control, whereas intracellular MRSA survival was inhibited after 6 h of infection in both cell lines (Fig. 8). Specifically, the effect of AMPK inhibition on MRSA intracellular survival was more pronounced in HUVECs than in HeLa cells. Furthermore, the levels of conversion of LC3-I to LC3-II were also significantly reduced in dorsomorphin-treated cells compared to nontreated cells after MRSA infection (Fig. 8C and D).

## DISCUSSION

### MRSA intracellular infection leads to extensive metabolic rerouting leading to the host exhibiting signs of metabolic starvation.

In this study, we were interested in the metabolic changes brought about by intracellular infection of nonphagocytic cells. We performed NMR and MS-based profiling and found that intermediates of lower glycolysis—such as phosphoenolpyruvate (PEP) and 3-phosphoglycerate (3-PG)—were highly increased in HeLa cells infected with the USA300 strain of S. aureus. Although the absolute levels of PEP and 3-PG were raised in HeLa cells after infection with the USA300 strain, the levels of lactate were relatively unaffected in both intracellular and extracellular compartments. Interestingly, the levels of intracellular pyruvate were increased in HeLa cells infected with the USA300 strain, and the secretion of this metabolite to the medium was also elevated in infected cells. This is suggestive of a downstream block explaining the accumulation of PEP and 3-PG. While the nature of this block is unknown, potential targets include (partial) inhibition of pyruvate kinase, the mitochondrial pyruvate carrier, and/or the pyruvate dehydrogenase complex, as well as regulatory interference of pyruvate dehydrogenase kinases.

In stark contrast, MRSA-infected cells excreted vastly increased quantities of acetate and formate. Acetate excretion is not uncommon in HeLa cell infection models as a product of bacterial activity (46), and our findings suggest elevated flux through bacterial pyruvate-formate lyase (Pfl). Pfl generates formate and acetyl coenzyme A (acetyl-CoA), which has been shown to be converted to acetate via phosphate acetyltransferase (Pta) and acetate kinase (AckA) for ATP generation (47) under some conditions. Furthermore, the increases in excretion (Fig. 1) and labeling patterns in USA300-infected cells (Fig. 3) for both formate and acetate are similar to each other and to those of pyruvate, suggesting a common source. These results suggest that despite higher glucose uptake in infected cells, bacterial metabolism might subvert some of the carbon intake, possibly contributing to a starvation response. While Pfl activity has mainly been activated under anaerobic conditions and in biofilms (48), intracellular Pfl activity might be a hallmark of intracellular *S. aureus* proliferation that warrants future investigation.

Among all the metabolic changes generally seen in host cells after an intracellular infection, alterations of the TCA cycle are relatively common (17, 49). The TCA cycle represents an important source of carbons and chemical energy for intracellular pathogens (18); therefore, it is likely that intracellular pathogens exploit this pathway for their intracellular survival and proliferation. In this study, the TCA cycle was greatly affected by MRSA infection, regardless of the strain or cell line employed. While glutamine was the main source of carbon for most of the intermediates, there was a clear rise in the glucose-derived label in fumarate and aspartate of infected cells. The reason for this difference in label distributions between fumarate/aspartate and succinate is not fully clear, but it could arise via several pathways. As the increase in glucose-derived label was mainly due to an increase in the M+3 ion (see Fig. S1 at https://figshare.com/s/e297ca2455dccb05307c), this suggests an anaplerotic flux from either pyruvate or PEP. One possibility is increased flux through pyruvate carboxylase forming oxaloacetate, potentially as a consequence of reduced flux through succinate dehydrogenase (Sdh), as previously observed in a study looking at the metabolic effects of loss-of-function *sdh* mutants (50). Moreover, the same study also found amino acid pool size alterations similar to those seen in the present study (50). Given the high levels of PEP, it is also possible that the reaction of PEP carboxykinase might be reversed: it usually catalyzes the decarboxylation of oxaloacetate to PEP during gluconeogenesis (51). On its own, however, this would not explain the difference between fumarate and malate labeling patterns. In addition, the increase in glutaminolysis that we have detected will increase the availability of ammonium, which can be used to convert oxaloacetate to aspartate, and this in turn is converted to fumarate in the urea cycle. Activation of the urea cycle is imminently plausible due to increased amounts of glutamate, an activator of *N*-acetylglutamate synthase, the product of which, *N*-acetylglutamate, is a key positive regulator of urea cycle activity (52).

### MRSA-induced starvation initiates an autophagy response that is exploited by the bacteria.

Among all its physiological functions, the role of autophagy as a defense against intracellular pathogens has been widely described (25, 27, 53, 54). However, intracellular pathogens have developed refined mechanisms to subvert autophagy/xenophagy and successfully survive within mammalian host cells (55–57). For instance, Salmonella enterica serovar Typhimurium and Mycobacterium tuberculosis inhibit the autophagy initiation signaling (58, 59), whereas Shigella flexneri escapes from autophagy recognition by recruiting Toca-1 via IcsB to inhibit its recognition by the autophagy machinery (60, 61). In contrast, S. aureus was shown to be able to replicate in autophagosomes and to eventually escape to the host cytosol (28, 62), although the molecular mechanisms behind this are not yet fully understood. However, our analysis revealed that a significant number of autophagosomes do not contain bacteria in MRSA-infected cells, suggesting that these autophagosomes may have been formed for an alternative purpose.

Autophagy inhibition with wortmannin impairs the intracellular proliferation of S. aureus, whereas the induction of autophagy by rapamycin results in an increased number of intracellular bacteria (28). On the other hand, autophagy confers cellular protection to the alpha-toxin produced by S. aureus (63). However, the alpha-toxin is not sufficient to permeabilize phagosomes in HeLa cells, probably reflecting differences in expression in the receptor of alpha-toxin monomers, the ADAM10 metalloprotease (9, 64, 65). Therefore, we hypothesize here that MRSA-free autophagosomes could be produced in response to a different autophagy-inducing stimulus than leaky phagosomes containing bacterial cells or the alpha-toxin produced by the pathogen.

The role of autophagy as a host defense pathway against intracellular pathogens has been already questioned, since the replication of some bacteria was impaired in autophagy-deficient cells and the treatment with autophagy activators recovered bacterial replication (66). Considering that autophagy could increase nutrient access, intracellular pathogens may have evolved some mechanisms to manipulate host autophagosomes and to exploit the products of autophagic degradation for bacterial survival and proliferation (22, 67). Intracellular MRSA causes multiple changes in cellular metabolism during infection, and many of the changes we observed point toward a situation of starvation, probably induced by the pathogen. This state of induced starvation might explain the presence of numerous MRSA-free autophagosomes.

In response to nutrient deprivation, autophagy is induced in mammalian cells (41, 44). This process is mainly regulated by mammalian target of rapamycin (mTOR), which acts as a sensor of the host metabolic state (68). In a wide range of mammalian cell lines, low levels of glucose enhance the autophagic machinery (44), whose regulation is usually dependent on AMPK signaling (69). During glucose deprivation, the proautophagy Beclin 1/VPS34 complex is activated via AMPK-dependent Beclin 1 phosphorylation (70). Moreover, a drop in the amino acid levels also induces autophagy in mammalian cells via the Ras/Raf-1/ErkI/II signaling pathway (44, 71). Additionally, in response to low levels of glucose, glutaminolysis is activated to sustain the TCA cycle and ATP production (44). As a consequence of the ammonia produced by the glutaminolysis pathway, the autophagic flux is increased (72, 73). In HeLa cells infected with the USA300 strain, all these starvation signals were present: reduction in the levels of glucose, a drop in several amino acids, and activation of glutaminolysis pathway. Interestingly, these changes were also observed in both HeLa cells and BALB/c mouse bone marrow macrophages infected with an alternative health care-associated MRSA (HA-MRSA) strain, suggesting that the results previously mentioned are not just strain and host dependent. In addition, we also observed activation of both AMPK and ERK signaling pathways in HeLa cells after MRSA infection. In accordance with these findings, a recent study has shown that the levels of ATP in human alveolar epithelial cells were significantly decreased during early time points of S. aureus infection (74), further pointing to a starvation-induced response. Crucially, once we inhibited the host AMPK pathway using dorsomorphin, intracellular MRSA survival was significantly hampered and autophagy levels were decreased, supporting our hypothesis that the activation of autophagy is caused by the state of starvation induced by the intracellular pathogen, and this autophagy benefits the intracellular survival of MRSA. Future studies need to be carried out to identify additional components of this regulatory pathway, but our findings suggest a novel host-directed antistaphylococcal strategy based on the inhibition of AMPK-mediated autophagy.

### Conclusions.

In this study, we have observed that autophagy may be activated in response to the metabolic state of the MRSA-infected host cell. During MRSA intracellular proliferation, the levels of glucose and several amino acids are significantly decreased in the host cell (likely due to bacterial metabolic activity), whereas glutaminolysis is highly activated. These observations are indicative of a state of starvation induced by the pathogen, which would explain the production of MRSA-free autophagosomes observed in infected host cells. This hypothesis is also supported by the activation of AMPK and ERK pathways, which act, respectively, as key sensors of cellular energy homeostasis and amino acid deprivation. In addition, we have identified AMPK as a new drug target for the development of alternative anti-infective compounds to traditional antibiotherapy against staphylococcal infection.

## MATERIALS AND METHODS

### Bacterial strains, cell lines, and culture conditions.

S. aureus USA300 LAC (75) and NCTC 13626 were cultured in nutrient broth (NB) (Sigma-Aldrich). Routinely, cells were grown for preinocula in 50-ml cultures to an optical density at 600 nm (OD_600_) of 1, centrifuged and washed with phosphate-buffered saline (PBS), and resuspended in 1.5 ml of PBS supplemented with 20% glycerol, and aliquots of 100 µl were stored at −80°C until further use. The CFU per milliliter of the preinocula was calculated by serial dilution plating.

HeLa cells (ECACC 93021013) were grown in Dulbecco’s modified Eagle’s medium (DMEM [Gibco]) containing pyruvate, glucose, and glutamine and supplemented with 10% heat-inactivated fetal bovine serum (FBS [Gibco]) and 5% penicillin and streptomycin solution (Gibco), unless otherwise stated. BALB/c mouse bone marrow macrophages (supplied by Calltag Medsystems) were cultured on complete macrophage medium supplemented with granulocyte-macrophage colony-stimulating factor, penicillin, streptomycin, and fetal bovine serum (Calltag Medsystems). Human umbilical vein endothelial cells (HUVECs [Sigma]) were grown in endothelial cell growth medium (Sigma).

HeLa cells expressing mCherry-LC3 (HeLa_mChe-LC3) were created by transduction (protocol from Boada-Romero et al. [76]). P12-MMP-mCherry-LC3 was kindly supplied by Felipe X. Pimentel Muiños (Centro de Investigación del Cancer de Salamanca, Spain). mCherry-LC3 was replaced with mCherry-CWT. Briefly, CWT was amplified from pLVTHM-Pm_YFPCWT (a kind gift from Martin Fraunholz, Würzburg, Germany) with primers GACGAGCTGTACAAGGGCTCGAGCCCATCA and TAGCTAGCGGCCGCTTTACTTTATAGTTCCCCAAAG, whereas mCherry was amplified with TAGCTAAAGCTTGCCACCATGGTGAGCAAGGGCGAG and TGATGGGCTCGAGCCCTTGTACAGCTCGTC, and subjected to fusion PCR. The resulting DNA amplicon was verified by sequencing, digested with HindIII/NotI, and cloned into P12-MMP.

For carbon labeling experiments for gas chromatography-mass spectrometry (GC-MS), minimum essential medium (MEM) containing 10% heat-inactivated FBS was supplemented with either unlabeled or universally ^13^C-labeled glucose or glutamine.

### Intracellular infection assay.

For metabolomics approaches, mammalian cells were seeded in 6-well plates (Sarstedt) in complete medium without antibiotics at a cell density of 5 × 10^5^ cells per well. The cells were infected after overnight adherence using 1 ml of fresh medium containing diluted S. aureus preinocula at a multiplicity of infection (MOI) of 100 and incubated at 37°C in 5% CO_2_ for 45 min. For the heat-killed condition, bacteria were incubated for 10 min at 95°C prior to centrifugation. After initial incubation, media were replaced by complete medium supplemented with 100 µg/ml gentamicin and cells were incubated for five additional hours, when the number of intracellular bacteria doubled while the host cell viability was halved (see Fig. S6A and B at https://figshare.com/s/e297ca2455dccb05307c). S. aureus NCTC 13626 is resistant to gentamicin (Fig. S6C); for that reason, gentamicin was replaced during NCTC 13626 infection assays with 5 µg/ml vancomycin, a clinically relevant antibiotic that is not active against intracellular S. aureus (32).

For host cell viability and bacterial survival, the intracellular infection assay was performed in 96- and 24-well plates, respectively. Host cell viability was quantified by flow cytometry with a fluorescein isothiocyanate (FITC)-annexin V apoptosis detection kit I (BD), following the manufacturer’s recommendations. Intracellular bacterial survival was quantified by lysing the cells with 100 µl of 0.1% Triton X-100 and quantifying CFU in agar plates.

### Metabolomics. (i) Extracellular metabolome analysis.

After 6 h of infection, medium was sampled and centrifuged (2,000 rpm, 5 min, 4°C), and the supernatant was stored immediately at −80°C. For analysis by nuclear magnetic resonance (NMR) spectroscopy, 0.8 ml of cell supernatant was mixed with 0.2 ml of NMR buffer (1 mM 4,4-dimethyl-4-silapentane-1-sulfonic acid [DSS] and 25 mM NaN_3_ in ^2^H_2_O, resulting in final concentrations of 0.2 and 5 mM, respectively). ^1^H NMR spectroscopy was performed on a Bruker DRX400 Avance spectrometer (Bruker Biospin, Rheinstetten, Germany) with a magnetic field strength of 9.4 T and a resonance frequency of 400 MHz. Spectra were acquired at 298 K into 32-K data points using a one-dimensional solvent suppression sequence, as described by Beckonert et al. (77).

### (ii) Intracellular metabolome analysis.

Cells were washed with 1 ml of ice-cold Ringer’s solution (Oxoid) after 6 h of infection, quenched by adding 1 ml of cold (−20°C) methanol, detached by using a cell scraper, and immediately stored at −80°C (78).

To obtain separated organic and aqueous fractions, extracts were dried using an Eppendorf Vacufuge concentrator, and a dual-phase extraction was performed by adding 300 µl of CHCl_3_/MeOH (2:1) and vortexing for 30 s. After addition of 300 µl of water and centrifugation (13,000 rpm, 10 min, room temperature), the top aqueous layer was transferred into an inactivated glass vial and dried before being stored at −80°C. The lower organic layers were placed into glass vials and dried overnight before being stored at −80°C.

For GC-MS, samples were derivatized by a two-step methoximation-silylation derivatization procedure. For normalization, a standard of 10 µl of 1.5 mg/ml myristic acid d27 standard solution (Sigma-Aldrich) was added and samples were dried. The dried samples were first methoximated using 20 µl of 20 mg/ml methoxyamine hydrochloride in anhydrous pyridine at 37°C for 90 min. For unlabeled samples, this was followed by silylation with 80 µl of *N*-methyl-*N*-(trimethylsilyl) trifluoroacetamide (MSTFA) at 37°C for 30 min (79). Labeled samples were derivatized by adding 80 µl of *N*-(tert-butyldimethylsilyl)-*N*-methyltrifluoroacetamide (MBTSFA [Thermo Scientific]). After being vortexed, samples were placed in a heater block at 70°C for 1 h. Eventually samples were centrifuged at 2,000 rpm for 5 min prior to transferring them into a clean vial for GC-MS analysis.

GC-MS analysis was performed on an Agilent 7890 gas chromatograph equipped with a 30-m DB-5MS capillary column with a 10-m Duraguard column connected to an Agilent 5975 MSD operating under electron impact ionization (Agilent Technologies UK, Ltd.). Samples were injected with an Agilent 7693 autosampler injector into deactivated splitless liners according to the method of Kind et al. (79), using helium as the carrier gas. Metabolites in the unlabeled pool were identified and quantified using a workflow described by Behrends et al. (80). Briefly, samples were deconvoluted in AMDIS (79, 81) and quantified using an in-house script. Integration of labeled metabolites was carried out based on an in-house fragment/retention time database using an updated version of the MatLab script capable of natural isotope correction (80, 82).

### Immunoproteomic approaches. (i) Cell extractions and protein lysates.

Cells were harvested by adding ice-cold phosphate-buffered saline (PBS) and detached by using a cell scraper (Thermo Fisher Scientific). Cell suspensions were transferred to 15-ml Falcon tubes and centrifuged (2,000 rpm, 10 min, 4°C). Cell pellets were washed with ice-cold PBS and immediately placed on ice for cell lysis. Lysis buffer (50 mM Tris [pH 7.5], 150 mM NaCl, 5 mM EDTA, 1% NP-40 detergent) was reconstituted with protease and phosphatase inhibitor cocktails (Sigma-Aldrich) and phenylmethylsulfonyl fluoride (PMSF [Sigma-Aldrich]), following the manufacturer’s instructions. Cell pellets were resuspended in 50 µl of lysis buffer, transferred to Eppendorf tubes, and incubated on ice for 30 min, applying heavy vortexing every 10 min. Then samples were centrifuged at maximum speed for 5 min at 4°C, and supernatants were transferred to new Eppendorf tubes and immediately stored at −80°C until needed.

The protein concentration was quantified by using the DC protein assay kit (Bio-Rad), and every cell lysate was adjusted to same concentration by diluting each sample in lysis buffer.

### (ii) SDS-PAGE.

Cell extractions were mixed with 2× reducing sample buffer (100 mM Tris [pH 6.8], 10% β-mercaptoethanol, 4% SDS, 0.3% bromophenol blue, 20% glycerol) and boiled in a heat block at 100°C for 5 min. Samples were loaded on polyacrylamide gels using 1× running buffer (25 mM Tris, 20 mM glycine, 0.05% SDS) and run on a Mini-Protean Tetra electrophoresis system (Bio-Rad) with a constant voltage of 100 V for 90 min.

### (iii) Western blotting and antibody detection.

Primary antibodies were purchased from Sigma (anti-β-actin [reference no. A5316]), Santa Cruz Biotechnologies (anti-GAPDH [reference no. sc-47724]), Fisher (anti-LC3 [reference no. 13278218]), and Cell Signalling Technology (anti-AMPK [reference no. 2793], anti-p-AMPK [reference no. 2535], anti-ERK [reference no. 4695], and anti-p-ERK [reference no. 5726]). Secondary antibodies were purchased from Li-Cor (IRDye 680LT goat anti-mouse [reference no. 926-68070] and IRDye 800LT goat anti-rabbit [reference no. 926-32211]).

Proteins were transferred onto nitrocellulose membranes (GE Healthcare) by using a Mini Trans-Blot cell (Bio-Rad) with 1× transfer buffer (25 mM Tris, 192 mM glycine, 20% methanol) and Whatman filter paper (GE Healthcare), following the manufacturer’s guidelines. The Western blot was run on the Tetra electrophoresis system at a constant voltage of 90 V for 2 h.

Nitrocellulose membranes were blocked using 5% skim milk diluted in Tris-buffered saline (TBS) solution (50 mM Tris [pH 7.6], 150 mM NaCl) for 1 h at room temperature. Once the blocking solution was removed, membrane was washed twice with TBS containing 0.05% Tween 20 (TBST) and subsequently incubated overnight at 4°C with the primary antibody. Following the suppliers’ recommendations, the primary antibodies were diluted to 1:1,000 in TBST containing 2% bovine serum albumin (BSA) and 0.1% sodium azide. After three washes with TBST, the membrane was incubated with the corresponding secondary antibody diluted 1:5,000 in 5% skim milk–TBST for 1 h at room temperature. The membrane was washed again three times with TBST and was developed employing the Odyssey Fc imaging system (Li-Cor). Images were taken, processed, and quantified by using Image Studio software (Li-Cor).

### Immunofluorescence microscopy.

HeLa cell lines expressing mCherry-LC3 were seeded in coverslips at a cell density of 7 × 10^4^ cells per well, and green fluorescent protein-expressing S. aureus strain USA300 (USA300-GFP) (83) was used to infect the cells at an MOI of 100. Infection was performed as previously described (76), and after 6 h of infection, cells were fixed with 0.5 ml of fresh 4% of paraformaldehyde (PFA) for 15 min. Coverslips were then washed twice with PBS and mounted on slides using ProLong Gold antifade mountant with DAPI (4′,6-diamidino-2-phenylindole [Thermo Fisher Scientific]) for nuclear staining.

Slides were observed on a confocal microscope Zeiss LSM 800 with Airyscan. Images were acquired and processed by using Zen Blue software (Zeiss).

### Statistical analysis.

Statistical analysis was conducted using GraphPad Prism software. Student’s *t* test and *post hoc* Tukey’s multiple-comparison tests were employed to examine significant differences across treatments.
